# A Non-Pharmacological Approach to Hip Osteoarthritis-Related Pain: A Case Report

**DOI:** 10.7759/cureus.109641

**Published:** 2026-05-25

**Authors:** Annie Chantasirivisal, Isabella N Lai, Qiuxue T Tan, Grant Chu

**Affiliations:** 1 Integrative Medicine, Infinity Wellness Institute, Los Angeles, USA; 2 Integrative Medicine, University of California Los Angeles, Los Angeles, USA; 3 Internal Medicine, University of California Los Angeles, Los Angeles, USA

**Keywords:** acupuncture, cupping, degenerative joint disease, hip osteoarthritis, multi-modality pain management, myofascial pain, trigger point injections

## Abstract

Hip osteoarthritis (OA) is a progressive degenerative joint condition associated with pain, functional limitation, and reduced quality of life in older adults. Standard management includes patient education, exercise therapy, and pharmacologic interventions, with surgical options reserved for advanced disease. However, symptom management is variable, particularly in patients with coexisting myofascial pain.

We present the case of a 67-year-old male with acute right hip pain following prolonged physical activity. Radiological findings confirmed structural degenerative changes consistent with hip OA-related pain and secondary myofascial pain. The patient underwent a multimodal treatment plan that consisted of acupuncture, trigger point injections (TPIs), and cupping therapy. Following a three-session treatment course, the patient reported significant pain reduction (VAS 10 to 2) and improved hip and lumbar range of motion. At 10-month follow-up, he reported sustained symptom resolution without recurrence or need for further intervention.

This case highlights the potential role of a targeted, multimodal approach addressing both osteoarthritic and myofascial contributors to pain. While each modality has demonstrated benefit individually, evidence supporting their combined application remains limited. Further controlled studies are warranted to evaluate the efficacy, reproducibility, and clinical role of such integrative strategies in the management of hip OA.

## Introduction

Osteoarthritis (OA) is a degenerative joint disease characterized by progressive cartilage loss, subchondral bone remodeling, and synovial inflammation, leading to pain and functional impairment [1]. Hip OA in particular is a major contributor to disability in older adults and is commonly associated with activity-related pain, reduced mobility, and decreased quality of life.

Current management strategies emphasize conservative care, including patient education, exercise therapy, and weight management, supplemented by pharmacologic interventions such as nonsteroidal anti-inflammatory drugs (NSAIDs). While these approaches are effective for many patients, symptom control remains variable, and some individuals experience persistent or recurrent pain despite standard treatment. In such cases, additional factors, including myofascial dysfunction, may contribute to symptom persistence and clinical complexity. As a result, non-pharmacological and multimodal strategies are increasingly explored to address persistent symptoms and the multifactorial nature of pain in hip OA, particularly when myofascial components are present. Multimodal and integrative treatment approaches have been increasingly explored to address both joint-related and soft tissue contributors to pain. However, evidence supporting the combined use of these therapies remains limited. 

This case report describes the clinical presentation, diagnostic considerations, and outcomes of a patient with acute hip OA-related pain and secondary myofascial involvement, who improved following a combined treatment approach incorporating acupuncture, trigger point injections (TPI), and cupping therapy.

## Case presentation

A 67-year-old male presented to an independent acupuncture clinic with acute, severe right hip pain that developed after two hours of continuous cycling. He had a history of previously manageable intermittent right hip pain after exercise. However, following the cycling event, he experienced a sudden onset of severe pain, limiting his ability to bear weight on the affected leg and disrupting sleep. His symptoms progressively worsened over three weeks, despite conservative measures including rest, warm baths, and over-the-counter ibuprofen. The patient described his pain as aching, sharp, and tingling, radiating down the lateral thigh, and rated it 10 out of 10 on the Visual Analog Scale (VAS). Pain worsened upon standing, lateral leg movement, prolonged weight-bearing, and lying down on his right side. He denied prior hip trauma or significant systemic illness; surgical history was notable only for an abdominal hernia repair. The patient was referred to physical therapy (PT) but discontinued treatment due to minimal improvement.

On physical examination, the patient demonstrated an antalgic gait with restricted hip movement due to pain. Pain was reproducibly elicited with hip flexion and internal rotation during examination. Palpation revealed tenderness over the greater trochanter and iliopsoas region, along with myofascial trigger points found in the right quadratus lumborum (QL); gluteal muscles (gluteus medius, gluteus minimus, and gluteus maximus); piriformis; tensor fasciae latae (TFL); biceps femoris (BF); and vastus lateralis (VL). Palpation of these trigger points reproduced the patient’s characteristic lateral thigh pain. Tenderness was assessed qualitatively based on patient-reported pain reproduction, and a formal grading scale was not utilized. 

Further examination revealed a reduced range of motion (ROM) in the hip and lumbar spine compared to normal values (Table 1). ROM was assessed by the clinician's visual estimation during routine physical examination without formal goniometric measurement. Patrick’s and Gaenslen’s tests were also performed and were negative bilaterally. A resisted straight-leg raise test did not reproduce radicular symptoms, and straight-leg raise testing was negative bilaterally. Neurological examination was grossly intact, with motor strength graded as 5/5 in the lower extremities, symmetric deep tendon reflexes (2+) at the patellar and Achilles tendons, and intact sensations to light touch. Additional neurological testing, including Tinel’s sign assessment and nerve conduction studies, was discussed during consultation but was not pursued by the patient.

**Table 1:** Hip and lumbar ROM at baseline and post-treatment. ROM: range of motion.

Measurement	Normal Reference	Baseline	Post-Treatment (1 Week)	Long-Term Follow-Up (10 Months)
Hip (right) – Flexion (°)	110–130	60	110	105
Hip (right) – Extension (°)	30	5	10	20
Lumbar – Flexion (°)	75	20	55	50
Lumbar – Extension (°)	30	10	30	30
Hip (right) – Abduction (°)	40–50	Limited	Improved Visual	40

**Figure 1:**
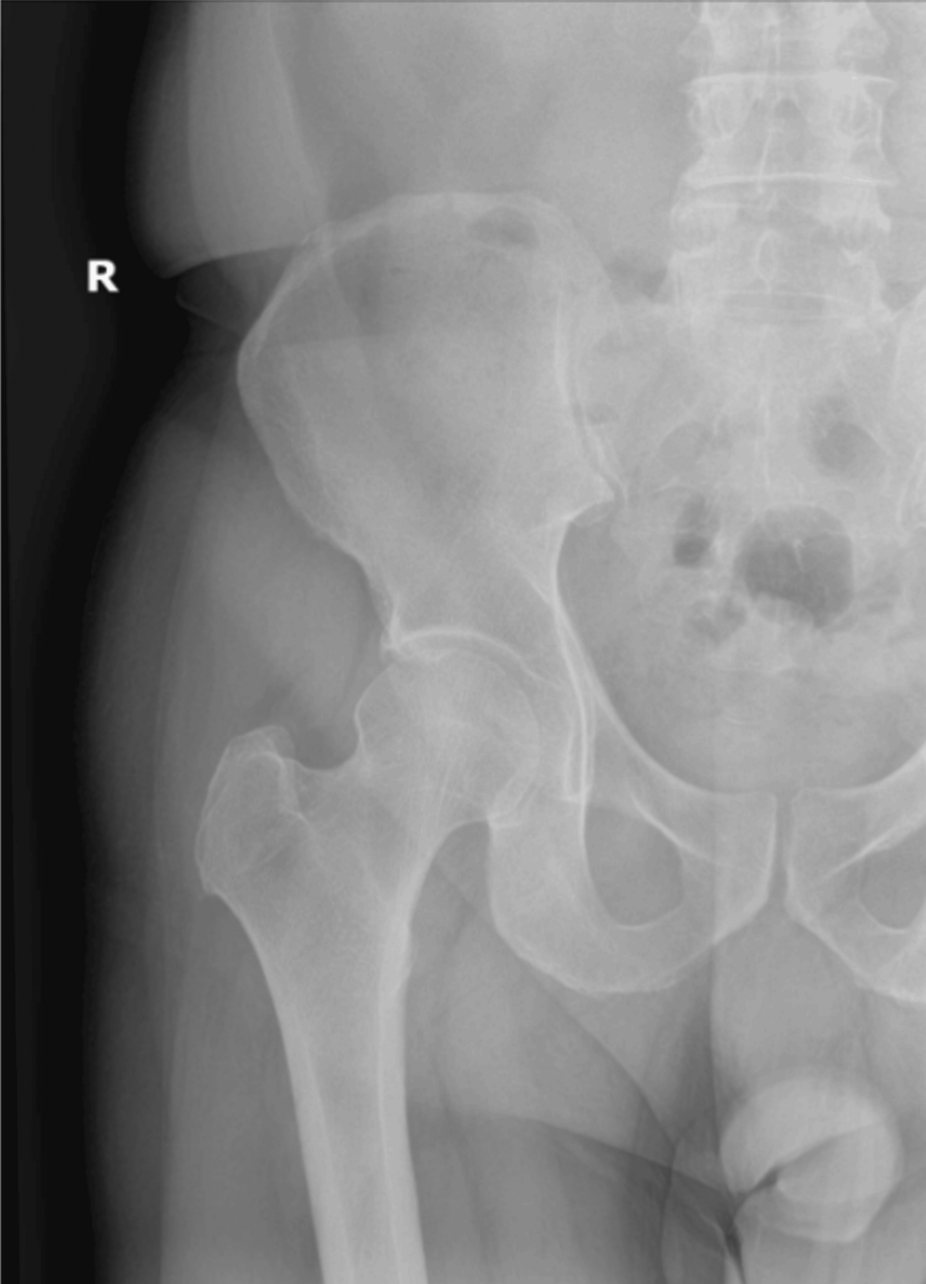
Right hip X-ray. The patient’s right hip X-ray indicates joint space narrowing and osteophyte formation consistent with moderate OA changes.

A right hip X-ray (Figure 1) showed narrowing of the joint space and osteophyte formation consistent with moderate OA changes; no evidence of fracture or avascular necrosis was found. MRI findings also revealed degenerative changes in the right hip joint. 

**Figure 2:**
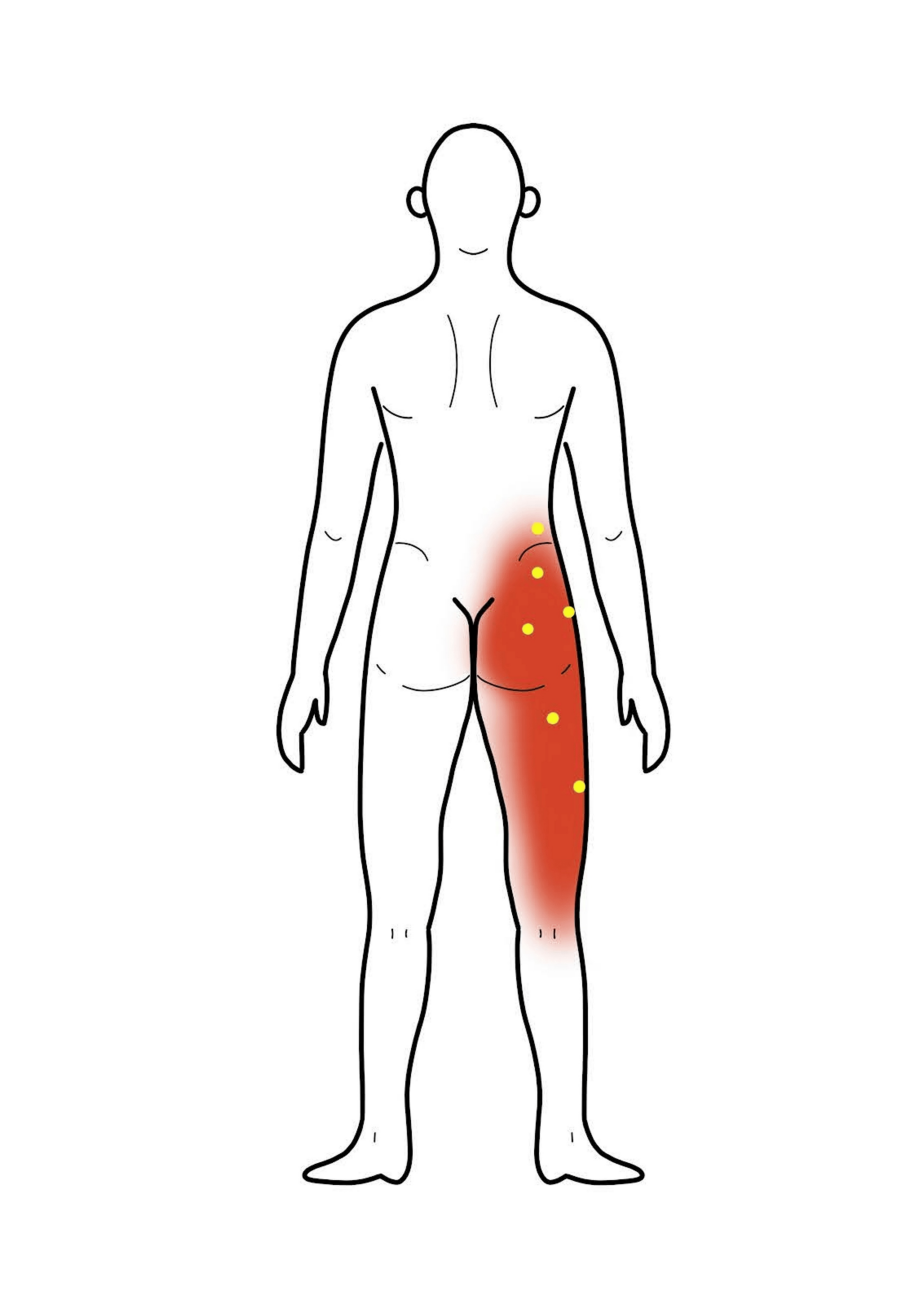
Location of administered TPIs. The patient described his right hip pain as radiating down the lateral thigh, illustrated by the shaded red areas. Tenderness associated with myofascial trigger points was observed in the right QL, gluteal muscles, piriformis, TFL, BF, and VL. TPIs were administered to these areas as denoted by the yellow points. This figure was created by the authors using Sketchbook.

The patient’s clinical presentation supported a diagnosis of right hip OA-related pain with secondary myofascial pain. Given the patient's severe pain and functional impairment, a multimodal pain management plan was implemented over three treatment sessions within one week. TPIs were administered to the right QL, gluteal muscles, piriformis, TFL, BF, and VL to relieve muscle spasms and referred pain, as shown in Figure 2. Each site received 0.5 mL of 1% lidocaine using a 25-gauge, 1.5-inch needle inserted at approximately 30 degrees to a depth of about 1 inch, with depth adjusted according to anatomical location and patient tolerance. 

Cupping therapy was applied to the right gluteal muscles, TFL, and VL for myofascial release, with suction titrated to patient comfort and clinical response. Cups were placed discontinuously for 15 minutes, at approximately 2-3 minutes per site. The acupuncture treatment was then administered using both local and distal points to address regional and systemic contributors to pain. Local points, including Gallbladder 29 (GB29, Ju Liao) and Gallbladder 30 (GB30, Huan Tiao), were selected to reduce lateral hip pain and enhance joint mobility. Distal points, including Spleen 8 (SP8, Diji), Stomach 40 (ST40, Fenglong), and Gallbladder 34 (GB34, Yang Ling Quan), were selected to alleviate lower extremity stiffness and weakness. Additionally, ashi points were targeted in areas of focal tenderness to address myofascial restriction and acute localized pain. Needles were retained for 30 minutes. The patient tolerated all interventions well, with no reported adverse or unanticipated events. Each one-hour treatment session included all three modalities, and three sessions were completed within one week.

In addition to procedural treatments, the patient also received education on adjunctive lifestyle modifications to support treatment outcomes. Recommendations included daily gentle stretching to maintain flexibility and reduce muscular tension, along with general anti-inflammatory dietary guidance as part of ongoing symptom management.

By the end of the three-session treatment course, the patient experienced symptom improvement, with pain reduced from 10 out of 10 to 2 out of 10 on the VAS. The patient also reported increased mobility, improved sleep, and the ability to bear weight without severe discomfort. Post-treatment physical exam revealed improvement of lumbar and hip ROM to near-normal values (Table 1). 

Ten months after completion of treatment, the patient returned to the clinic for evaluation of a new complaint involving right shoulder pain. During this visit, he reported sustained resolution of his right hip pain, with pain remaining at 2 out of 10 on the VAS, no recurrence of hip OA-related functional limitations, and a return to regular daily activities without the need for assistive devices (Table 2). Management during this visit was therefore directed toward the new shoulder complaint.

**Table 2:** Clinical and functional outcomes at baseline, post-treatment, and long-term follow-up. VAS: Visual Analog Scale; ADLs: activities of daily living.

Functional Outcome	Initial Presentation (Baseline)	Post-Treatment Follow-Up (1 Week)	Long-Term Follow-Up (10 Months)
Pain (VAS, 0–10)	10	2	2
Sleep disturbance	Present (nightly)	Improved	None reported
Weight-bearing status	Unable to bear weight	Able to stand/walk with mild pain	Fully returned to baseline
ADLs	Severely limited	Improved	Normal

Given the patient’s presentation of lateral thigh pain and lower extremity discomfort, the differential diagnosis included sacroiliac joint (SI) dysfunction, lumbar radiculopathy, sciatica, and iliotibial band (ITB) syndrome. Based on clinical findings, imaging, and neurological evaluation, these conditions were considered less likely, and a working diagnosis of hip OA-related pain with secondary myofascial involvement was made (Table 3). The patient’s acute presentation, combined with altered biomechanics, may have contributed to compensatory overuse of surrounding musculature, resulting in secondary myofascial dysfunction and trigger point activation. The clinical course of presentation, intervention, and outcomes is summarized in a timeline (Table 4).

**Table 3:** Differential diagnosis and clinical findings supporting final diagnosis. SI: sacroiliac; SLR: straight leg raise; EMG: electromyography; NCS: nerve conduction studies; ITB: iliotibial band; OA: osteoarthritis; ROM: range of motion.

Differential Diagnosis	Key Tests/Findings	Findings in This Case
SI joint dysfunction	FABER/Patrick’s, Gaenslen’s, SI palpation	FABER and Gaenslen’s negative; no tenderness over SI joints
Lumbar radiculopathy (L3–L5)	Dermatomal pain/paresthesia, reflex/motor deficits, SLR/slump positive; consider EMG/NCS	Neurological exam grossly intact; no dermatomal deficits; SLR negative at 70° bilaterally; no reproduction of radicular symptoms; advance neurodiagnostic testing not pursued
Sciatica	SLR/slump positive, radicular distribution	Negative SLR; pain distribution non-dermatomal and localized to lateral thigh; no reproduction of radicular pain
ITB syndrome	Ober’s test positive; lateral knee pain	Negative Ober’s test; no lateral knee pain; no reproduction of characteristic ITB pain
Hip OA	Radiographic joint-space narrowing/osteophytes; pain with ROM, morning stiffness	Consistent with radiographic findings; pain reproduced with hip flexion and internal rotation

**Table 4:** Timeline of clinical course. VAS: Visual Analog Scale.

Timepoint	Event
Week 0	Acute onset of right hip pain following prolonged cycling
Weeks 1-3	Progressive worsening of pain and functional limitation
Beginning of Week 4	Presentation to clinic and initiation of treatment
Week 4	Completion of three treatment sessions
Beginning of Week 5	Marked improvement in pain (VAS from 10/10 to 2/10) and function
10-month follow-up	Sustained symptom improvement without recurrence or need for further intervention

## Discussion

Hip OA is a degenerative joint condition in which cartilage gradually breaks down due to biomechanical stress, overuse, and aging; symptoms often include pain localized to the hip and groin [1]. Although physical examination may suggest hip OA, imaging can help confirm the diagnosis and assess structural changes such as joint space narrowing, osteophyte formation, and subchondral sclerosis [1]. Hip OA is increasingly prevalent with age: approximately 5-15% of individuals over 55 may develop symptomatic OA, rising to 25% in those over 85; nearly 10% later undergo total hip arthroplasty [2-4]. Notably, symptom severity may fluctuate over time, independent of structural progression, which can complicate interpretation of treatment-related outcomes. 

Recent literature suggests broadly similar treatment plans across OA subtypes, including hip OA; however, studies specifically focused on hip OA management remain limited [5]. First-line conservative management typically includes patient education, structured exercise therapy, and weight management, which have demonstrated improvements in pain and functional outcomes in mild-to-moderate OA of the hip and knee [6]. Within pharmacologic management, NSAIDs and other analgesics are commonly used, although effectiveness varies. One study reported that approximately 70% of patients used prescription medications and 44% to 70% used over-the-counter analgesics; among these individuals, 72% reported symptomatic relief without requiring additional interventions [7]. However, limitations including dose-related tolerance, transient analgesic effects, and adverse effects associated with prolonged use have also been reported [8]. Physical therapy (PT) has shown similarly variable outcomes; a randomized controlled trial found no significant difference between active PT and sham intervention, while another review suggests PT may be comparable to NSAIDs and superior to acetaminophen in improving pain and function in mild to moderate OA [9,10]. Surgical intervention, such as total hip arthroplasty, is generally reserved for advanced or refractory cases due to associated risks and is not considered first-line therapy, particularly in older adults [11].

This case describes a multimodal approach combining acupuncture, TPIs, and cupping therapy, each with proposed complementary mechanisms. Acupuncture has been shown to modulate pain perception through vagal and somato-autonomic pathways, increasing endogenous opioid release and decreasing pro-inflammatory cytokines [12,13]. While some studies show limited benefit over sham treatments, trials with larger sample sizes show symptom improvements when combined with routine care [14-16]. TPIs can also manage myofascial pain; one study found that dry needling (DN) improved pain and ROM in hip OA, while another study concluded lidocaine-based TPIs to be superior to hyaluronic acid injections for knee OA [17,18]. Cupping therapy has also demonstrated improvements in local circulation, pain, and soft tissue mobility in knee OA, although direct evidence in hip OA remains limited [19]. 

Evidence supporting the combined use of these modalities remains limited. However, given the complexity and multifactorial pathophysiology of myofascial pain, a multimodal, patient-centered approach may be clinically appropriate. Prior studies have demonstrated benefit for individual interventions, including TPIs, DNs, and manual therapies, when compared to sham treatments, though standardized treatment algorithms are lacking and no single first-line therapy is universally effective. Conventional approaches, including physical therapy, NSAIDs, and surgical intervention, may provide variable or incomplete relief, particularly when myofascial dysfunction contributes to symptom persistence [20]. In this context, the combined use of multiple evidence-supported modalities in a complementary manner represents an exploratory clinical strategy. This case contributes to the emerging body of literature by demonstrating a favorable clinical response while underscoring the need for controlled studies to evaluate potential synergistic effects and clarify its role relative to current conventional treatments.

This case report is limited by its single-patient design, which restricts generalizability and precludes comparison with alternative interventions. Diagnostic interpretation was complicated by overlapping features of hip osteoarthritis and other musculoskeletal or neuropathic conditions. Although neurological examination findings were unremarkable, advanced neurodiagnostic testing, including electromyography and nerve conduction studies, was not performed, as further evaluation was deferred by the patient, limiting complete exclusion of neuropathic etiologies. Outcomes were primarily based on patient-reported pain measures and clinician-estimated range of motion, which may introduce subjectivity and reduce reproducibility. Additionally, objective post-treatment validation through imaging or standardized functional assessments was not obtained. Implementation of a multimodal approach may vary depending on clinical setting, practitioner expertise, and available resources. While procedural details have been described, the multimodal treatment approach was individualized and not based on a standardized protocol, which may limit reproducibility across different clinical settings. Although follow-up at 10 months demonstrated sustained symptom improvement, the absence of systematic longitudinal assessment limits evaluation of long-term durability. 

Despite these limitations, this case highlights the diagnostic value of a detailed musculoskeletal examination in differentiating secondary myofascial pain from primary joint pathology in hip OA. It demonstrates a pragmatic, patient-centered application of a multimodal approach in a complex presentation, integrating therapies with complementary mechanisms of action. The case also contributes to the limited literature on combined-modality use in hip OA with associated myofascial pain and includes extended follow-up, offering insight into the potential durability of clinical improvement.

## Conclusions

This case describes a multimodal approach incorporating acupuncture, TPIs, and cupping therapy for managing acute hip OA-related pain with secondary myofascial dysfunction. Although initial presentation raised concerns for neurogenic etiologies, the patient’s response to localized treatment was more consistent with musculoskeletal etiology. Existing literature has demonstrated benefit for these modalities individually; however, evidence supporting their combined use remains limited. The observed clinical improvement suggests that such an approach may be beneficial in select patients, but given the inherent limitations of a single case, these findings should be interpreted with caution. Further controlled studies are needed to evaluate the efficacy, reproducibility, and potential role of this multimodal strategy in relation to established standard-of-care treatments and to better define its role within evidence-based management of hip OA.

## References

[1] Murphy NJ, Eyles JP, Hunter DJ (2016). Hip osteoarthritis: etiopathogenesis and implications for management. Adv Ther.

[2] Kloek CJ, Bossen D, Spreeuwenberg PM (2018). Effectiveness of a blended physical therapist intervention in people with hip osteoarthritis, knee osteoarthritis, or both: a cluster-randomized controlled trial. Phys Ther.

[3] Murphy LB, Helmick CG, Schwartz TA (2010). One in four people may develop symptomatic hip osteoarthritis in his or her lifetime. Osteoarthritis Cartilage.

[4] Culliford DJ, Maskell J, Kiran A (2012). The lifetime risk of total hip and knee arthroplasty: results from the UK general practice research database. Osteoarthritis Cartilage.

[5] Hermann W, Lambova S, Muller-Ladner U (2018). Current treatment options for osteoarthritis. Curr Rheumatol Rev.

[6] Sinatti P, Sánchez Romero EA, Martínez-Pozas O, Villafañe JH (2022). Effects of patient education on pain and function and its impact on conservative treatment in elderly patients with pain related to hip and knee osteoarthritis: a systematic review. Int J Environ Res Public Health.

[7] Driban JB, Boehret SA, Balasubramanian E (2012). Medication and supplement use for managing joint symptoms among patients with knee and hip osteoarthritis: a cross-sectional study. BMC Musculoskelet Disord.

[8] Selten EM, Vriezekolk JE, Geenen R (2016). Reasons for treatment choices in knee and hip osteoarthritis: a qualitative study. Arthritis Care Res (Hoboken).

[9] Bennell KL, Egerton T, Martin J (2014). Effect of physical therapy on pain and function in patients with hip osteoarthritis: a randomized clinical trial. JAMA.

[10] Skou ST, Roos EM (2019). Physical therapy for patients with knee and hip osteoarthritis: supervised, active treatment is current best practice. Clin Exp Rheumatol.

[11] Hamel MB, Toth M, Legedza A, Rosen MP (2008). Joint replacement surgery in elderly patients with severe osteoarthritis of the hip or knee: decision making, postoperative recovery, and clinical outcomes. Arch Intern Med.

[12] Zhao ZQ (2008). Neural mechanism underlying acupuncture analgesia. Prog Neurobiol.

[13] Lin JG, Chen WL (2008). Acupuncture analgesia: a review of its mechanisms of actions. Am J Chin Med.

[14] Manheimer E, Cheng K, Wieland LS (2018). Acupuncture for hip osteoarthritis. Cochrane Database Syst Rev.

[15] Fink MG, Kunsebeck H, Wipperman B, Gehrke A (2001). Non-specific effects of traditional Chinese acupuncture in osteoarthritis of the hip. Complement Ther Med.

[16] Witt CM, Jena S, Brinkhaus B (2006). Acupuncture in patients with osteoarthritis of the knee or hip: a randomized, controlled trial with an additional nonrandomized arm. Arthritis Rheum.

[17] Ceballos-Laita L, Jiménez-Del-Barrio S, Marín-Zurdo J (2019). Effects of dry needling in HIP muscles in patients with HIP osteoarthritis: A randomized controlled trial. Musculoskelet Sci Pract.

[18] Yentür EA, Okçu G, Yegül I (2003). The role of trigger point therapy in knee osteoarthritis. Pain Clin.

[19] Li JQ, Guo W, Sun ZG (2017). Cupping therapy for treating knee osteoarthritis: The evidence from systematic review and meta-analysis. Complement Ther Clin Pract.

[20] Steen JP, Jaiswal KS, Kumbhare D (2025). Myofascial pain syndrome: an update on clinical characteristics, etiopathogenesis, diagnosis, and treatment. Muscle Nerve.

